# Supplementary material for: Climatic niche conservatism in non-native plants depends on introduction history and biogeographic context
Source: Nat Commun. 2026 Jan 9;17:416. doi: 10.1038/s41467-025-68023-6 (PMC12796159; doi:10.1038/s41467-025-68023-6)
Supplement: Supplementary file 1 — Supplementary Information [file 41467_2025_68023_MOESM1_ESM.pdf]

Supplementary material: Climatic niche conservatism in non-native plants depends on introduction history and biogeographic context.

Anna Rönnfeldt[1,*], Valén Holle[1], Katrin Schifferle[1], Laure Gallien[2], Tiffany Knight[3,4,5,6], Patrick Weigelt[7,8], Dylan Craven [9,10], Juliano Sarmento Cabral[11,12], Damaris Zurell[1]

Affiliations:

[1] University of Potsdam, Inst. for Biochemistry and Biology, D-14469 Potsdam, Germany
[2] University Grenoble Alpes, University Savoie Mont Blanc, CNRS, LECA, Laboratoire d'Ecologie Alpine, F-38000 Grenoble, France
[3] Department of Species Interaction Ecology, Helmholtz Centre for Environmental Research – UFZ, Permoserstraße 15, Leipzig 04318, Germany
[4] German Centre for Integrative Biodiversity Research (iDiv), Halle-Jena-Leipzig, Puschstrasse 4, Leipzig 04103, Germany
[5] Institute of Biology, Martin Luther University Halle-Wittenberg, Am Kirchtor 1, 06108, Halle (Saale), Germany
[6] German Centre for Integrative Biodiversity Research (iDiv), Halle-Jena-Leipzig, Puschstrasse 4, Leipzig 04103, Germany Department of Science and Conservation, National Tropical Botanical Garden, Kalāheo, HI, USA
[7] Department of Environmental Science, Radboud Institute for Biological and Environmental Sciences (RIBES), Radboud University, Heyendaalseweg 135, 6525AJ Nijmegen, The Netherlands.
[8] Biodiversity, Macroecology & Biogeography, University of Göttingen, Büsgenweg 1, 37077 Göttingen, Germany
[9] GEMA Center for Genomics, Ecology & Environment, Universidad Mayor, Camino La Pirámide 5750, Huechuraba, Santiago, Chile
[10] Data Observatory Foundation, ANID Technology Center No. DO210001, Eliodoro Yáñez 2990, 7510277, Providencia, Santiago, Chile
[11] University of Birmingham, College of Life and Environmental Sciences, School of Biosciences, B15 2TT Birmingham, UK
[12] Ecological Modelling, Bonner Institute for Organismal Biology - Dept. of Plant Biodiversity, University of Bonn, Bonn, Germany

* Corresponding author: anna.roennfeldt@uni-potsdam.de

Table of Contents

Supplementary Figures 3
Map of the study regions (Figure S1) 3
Species flow from native main climate zones to non-native regions (Figure S2) 4
Similarity test outcomes showing the regional split in percentage (Figure S3) 5
Niche dynamics across analogue and non-analogue niche space (Figure S4) 6
Buffer sensitivity: Regional niche conservatism (Figure S5) 7
Buffer sensitivity: Regional niche unfilling (Figure S6) 8
Buffer sensitivity: Regional niche stability (Figure S7) 9
Buffer sensitivity: Regional niche expansion (Figure S8) 10
Mean trait values for species from different climate zones (Figure S9) 11
Trait analysis: results for abandonment and pioneering (Figure S10) 12
Trait analysis: full models for abandonment and pioneering (Figure S11) 13
Trait analysis: univariate models for all niche metrics (Figure S12) 14
Supplementary Tables 15
ANOVA table for the comparison of the regional niche dynamics (Table S1) 15
Phylogenetic signals in the trait analyses (Table S2) 16
R packages used for the analyses 17
References 19

Supplementary Figures

Map of the study regions (Figure S1)

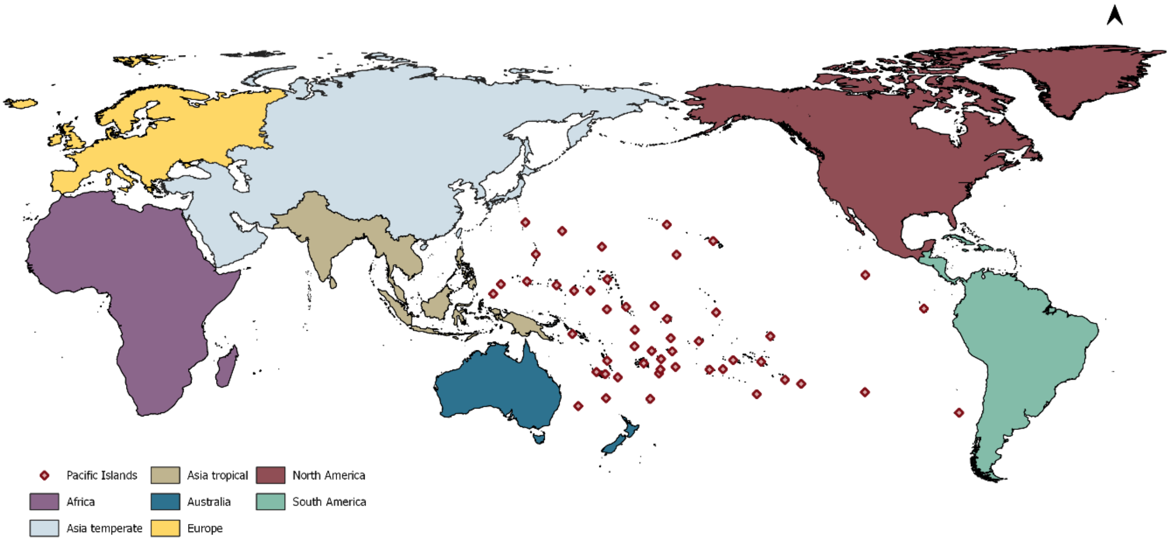

Figure S1. Map of the eight study regions. The regions correspond to level 1 of the World Geographic Scheme for Recording Plant Distributions[1], except for the Pacific Islands which are based on Wohlwend et al.[2] and consist of a subset of 50 island groups, with a red rhombus highlighting the center of each island group, respectively.

Species flow from native main climate zones to non-native regions (Figure S2)

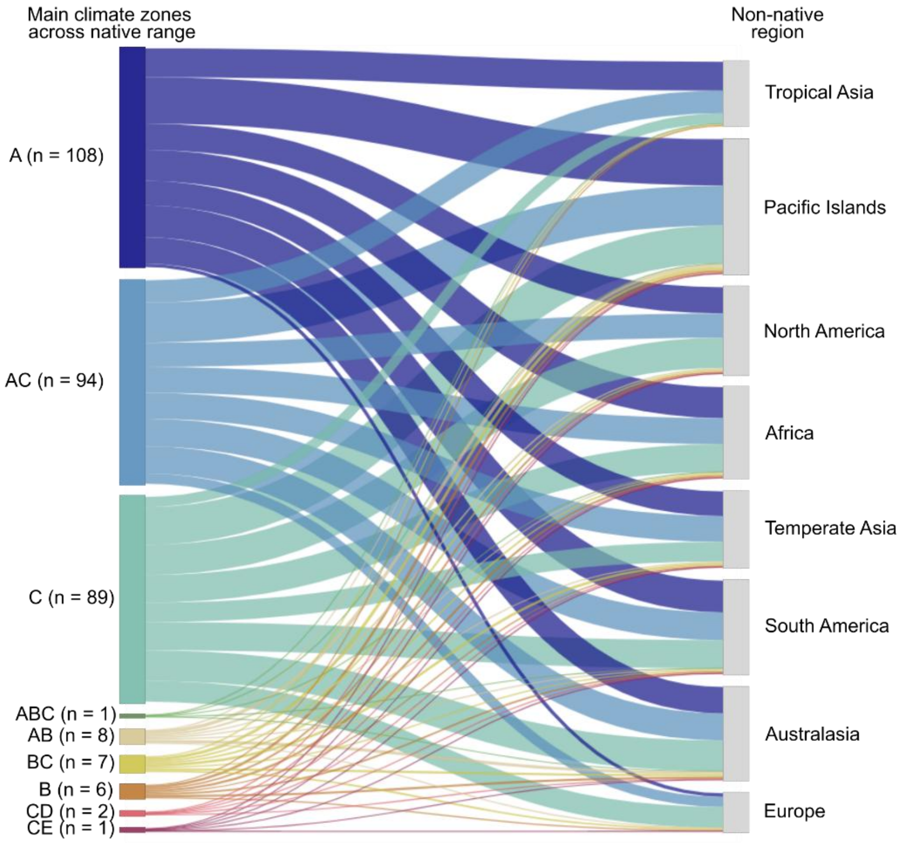

Figure S2 – Species flows from native main climate zones to non-native regions. The climate zones are based on the main climate zones of the Köppen-Geiger climate classification[3]: A – tropical, B – arid, C – temperate, D – continental, E – polar. A climate zone was considered to be among the main climate zones for a species if 30 % of the species' native occurrences lie within that zone. The number of species associated with the respective climate zones is given in the brackets behind the labels. Source data are provided as a Source Data file.

Similarity test outcomes showing the regional split in percentage (Figure S3)

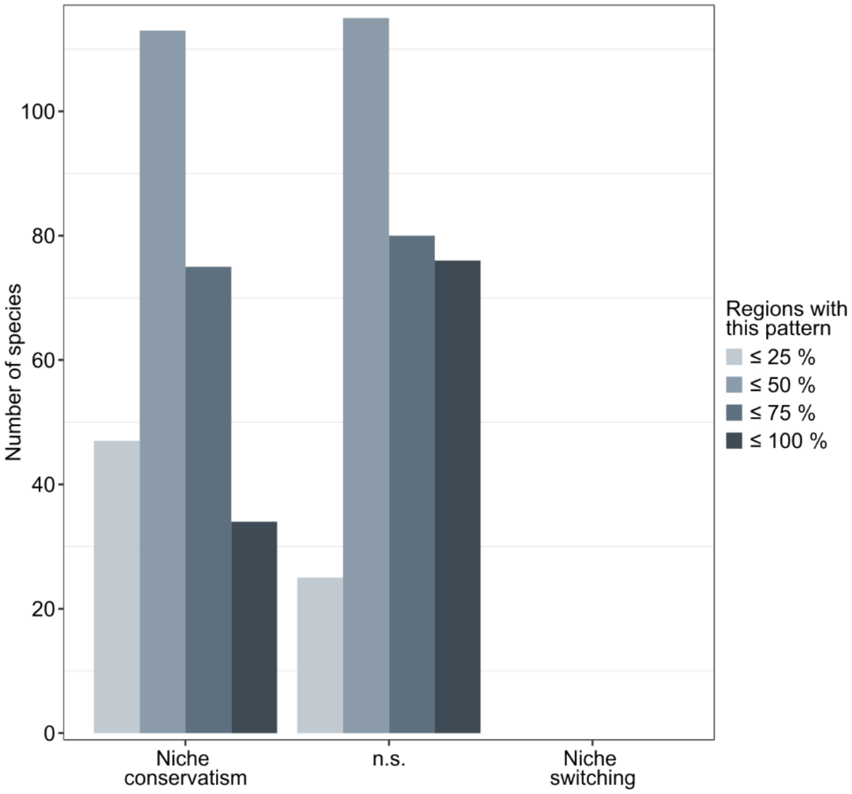

Figure S3 - Number of species showing climatic niche conservatism or switching. Significant niche conservatism and switching were determined with similarity tests (n = 1200 iterations). The colored bars indicate the number of species that consistently showed the respective outcomes in x percentage of the regions they have been introduced to. Source data are provided as a Source Data file.

Niche dynamics across analogue and non-analogue niche space (Figure S4)

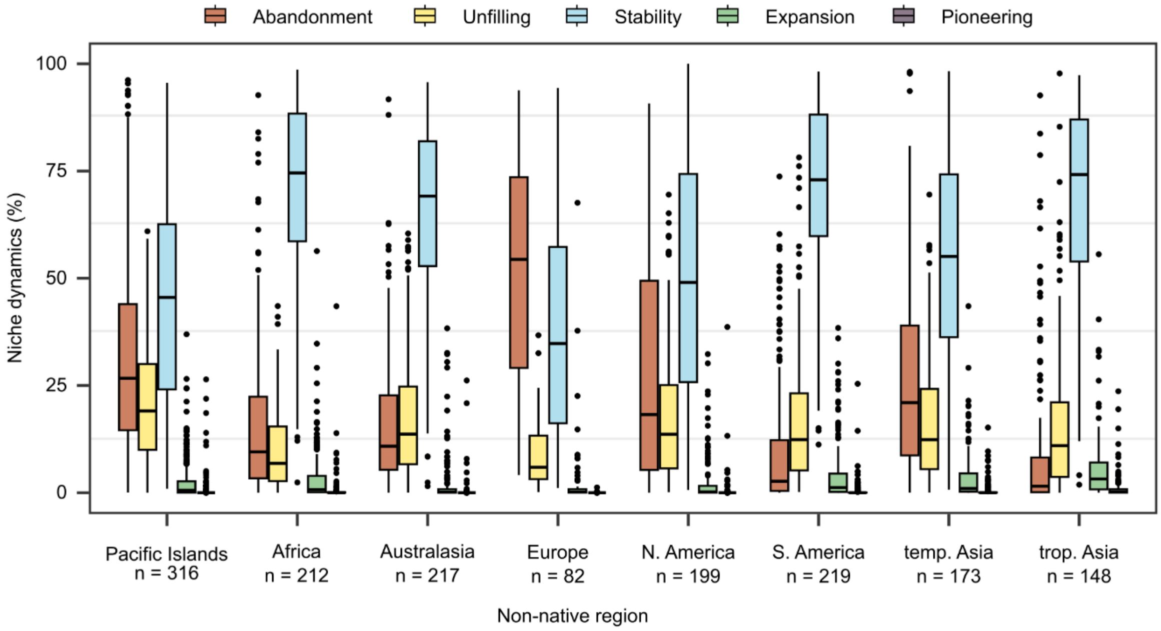

Figure S4 – Niche dynamics in the non-native ranges. The box outlines indicate the interquartile range (IQR), and the horizontal lines represent the median values. The whiskers extend no more than 1.5 times the IQR from the boxes, with individual points identified as outliers. The sample size under each region name indicates the number of species that have been introduced to that region. Source data are provided as a Source Data file.

Buffer sensitivity: Regional niche conservatism (Figure S5)

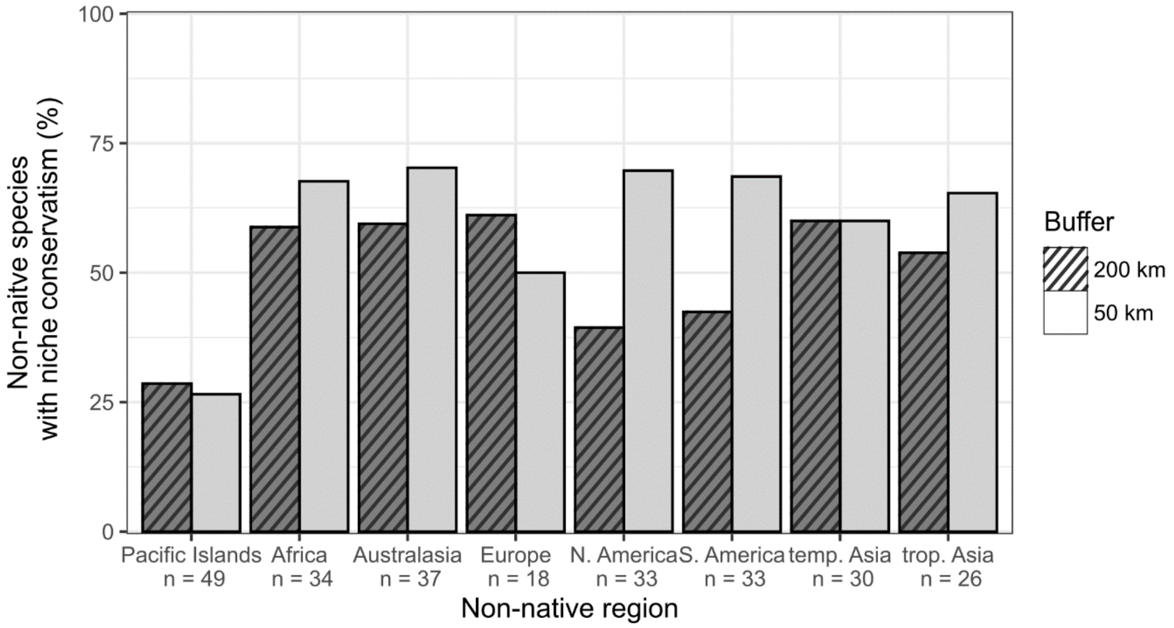

Figure S5 – Regional percentage of species that conserved their niche for different buffer sizes. Significant niche conservatism was determined with similarity tests (n = 1200 iterations). Background data were sampled within a 200 km (striped pattern) or 50 km (no pattern) buffer around the presence points. The sample size under each region name indicates the number of species that have been introduced to that region. Source data are provided as a Source Data file.

Buffer sensitivity: Regional niche unfilling (Figure S6)

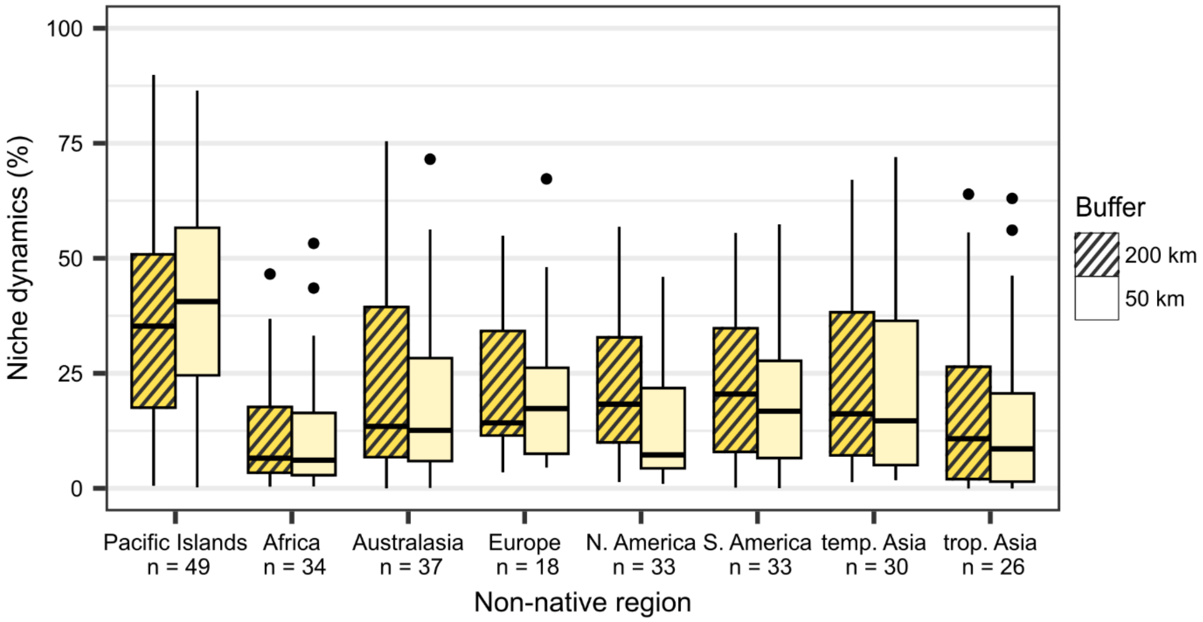

Figure S6 – Niche unfilling in the non-native ranges for different buffer sizes. Background data were sampled within a 200 km (striped pattern) or 50 km (no pattern) buffer around the presence points. The sample size under each region name indicates the number of species that have been introduced to that region. Source data are provided as a Source Data file.

Buffer sensitivity: Regional niche stability (Figure S7)

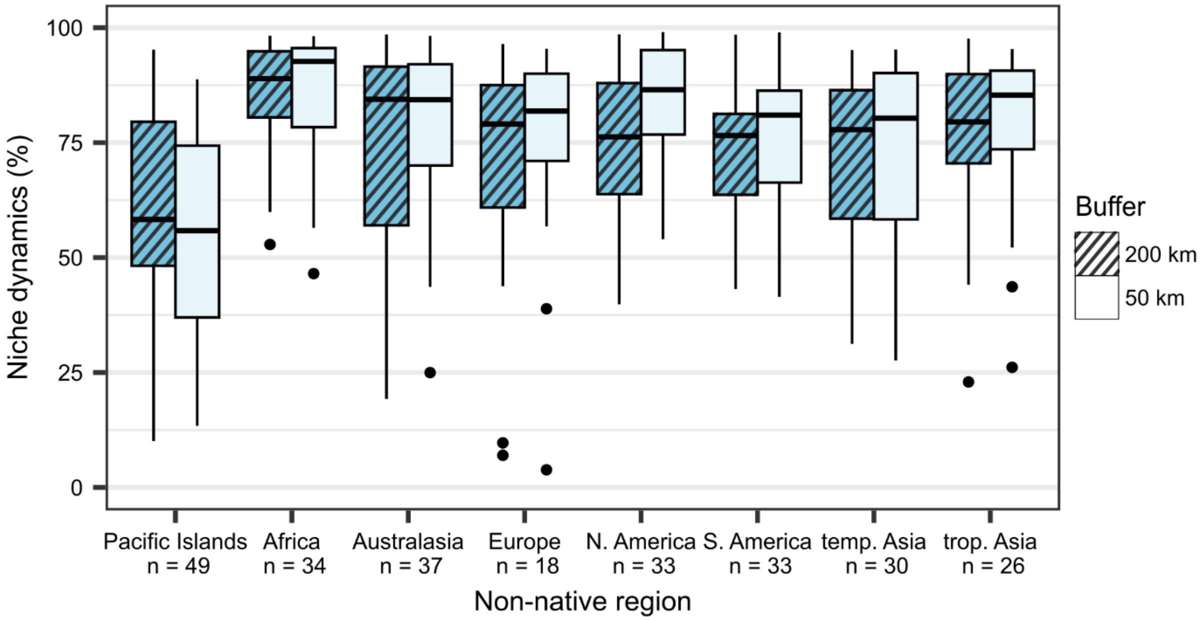

Figure S7 – Niche stability in the non-native ranges for different buffer sizes. Background data were sampled within a 200 km (striped pattern) or 50 km (no pattern) buffer around the presence points. The sample size under each region name indicates the number of species that have been introduced to that region. Source data are provided as a Source Data file.

Buffer sensitivity: Regional niche expansion (Figure S8)

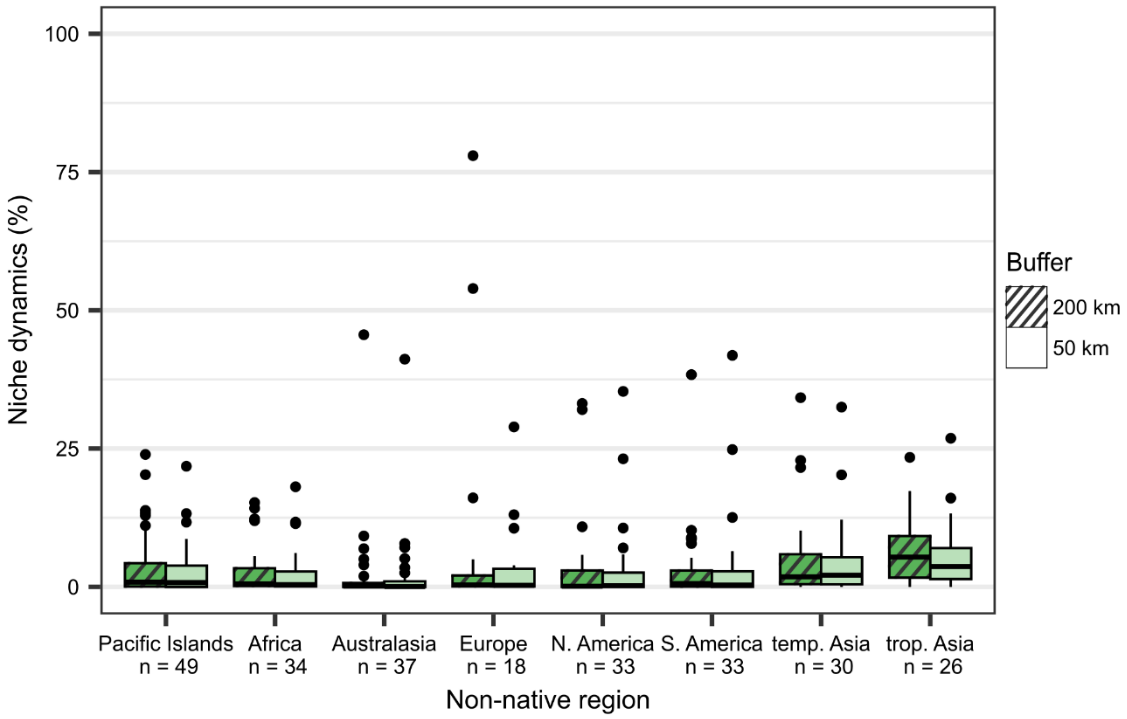

Figure S8 – Niche expansion in the non-native ranges for different buffer sizes. Background data were sampled within a 200 km (striped pattern) or 50 km (no pattern) buffer around the presence points. The sample size under each region name indicates the number of species that have been introduced to that region. Source data are provided as a Source Data file.

Mean trait values for species from different climate zones (Figure S9)

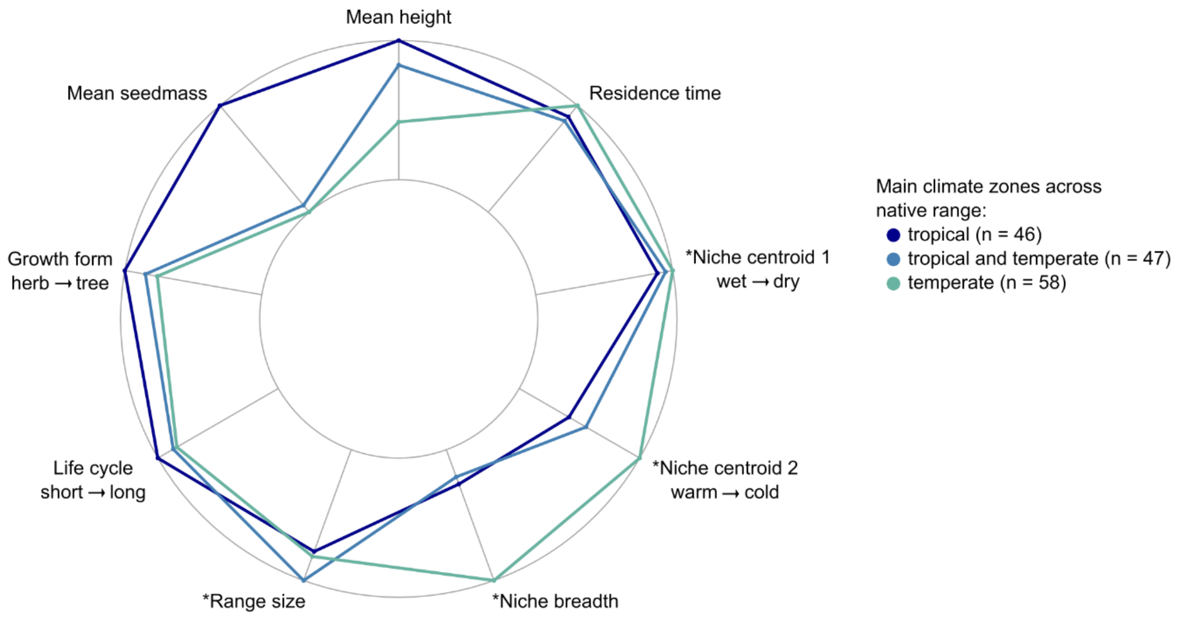

Figure S9 – Mean trait values for species native to different climate zones. Traits labelled with an asterisk refer to biogeographic traits estimated for the native range or native niche of the species. Source data are provided as a Source Data file.

Trait analysis: results for abandonment and pioneering (Figure S10)

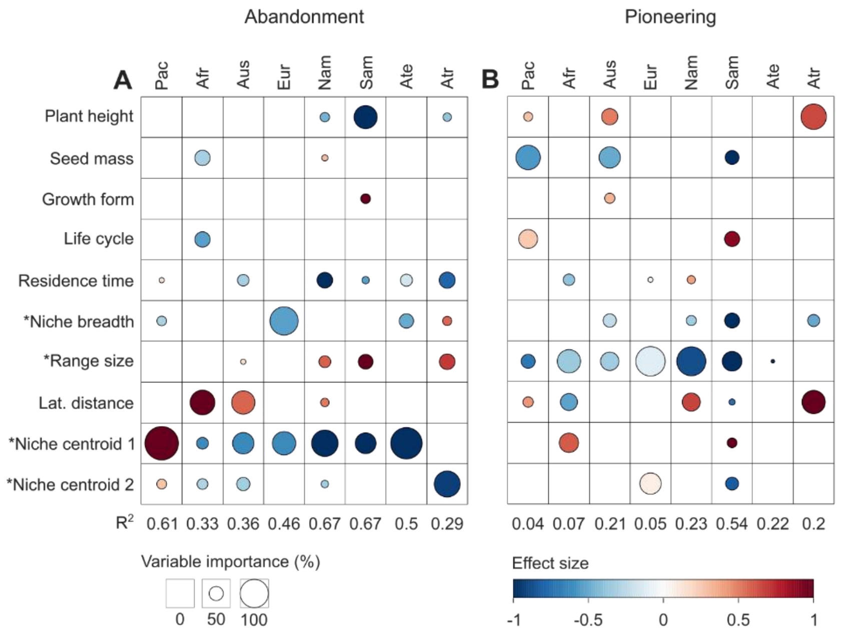

Figure S10 – Trait effects on the non-analogue niche change metrics in non-native plants. For each region, we ran AIC-based stepwise phylogenetic regression models with (A) niche abandonment and (B) niche pioneering as responses. The final, parsimonious models per region are shown in each column, with the total explained variance (R^2) of the models shown below the columns. Circle sizes indicate the variable importance (%) of single traits within the multiple regression models, and the effect size shows whether the respective niche change metrics will increase (red) or decrease (blue) as the trait values increase. Traits labelled with an asterisk refer to biogeographic traits estimated for the native range or native niche of the species. Niche centroids refer to the relative position along climatic gradient 1 (from warm to cold) and 2 (from wet to dry). The species sample size varied between regions: Africa (Afr, n = 124), temperate Asia (Ate, n = 95), tropical Asia (Atr, n = 78), Australasia (Aus, n = 124), Europe (Eur, n = 56), North America (Nam, n = 110), Pacific Islands (Pac, n = 143), South America (Sam, n = 41). Source data are provided as a Source Data file.

Trait analysis: full models for abandonment and pioneering (Figure S11)

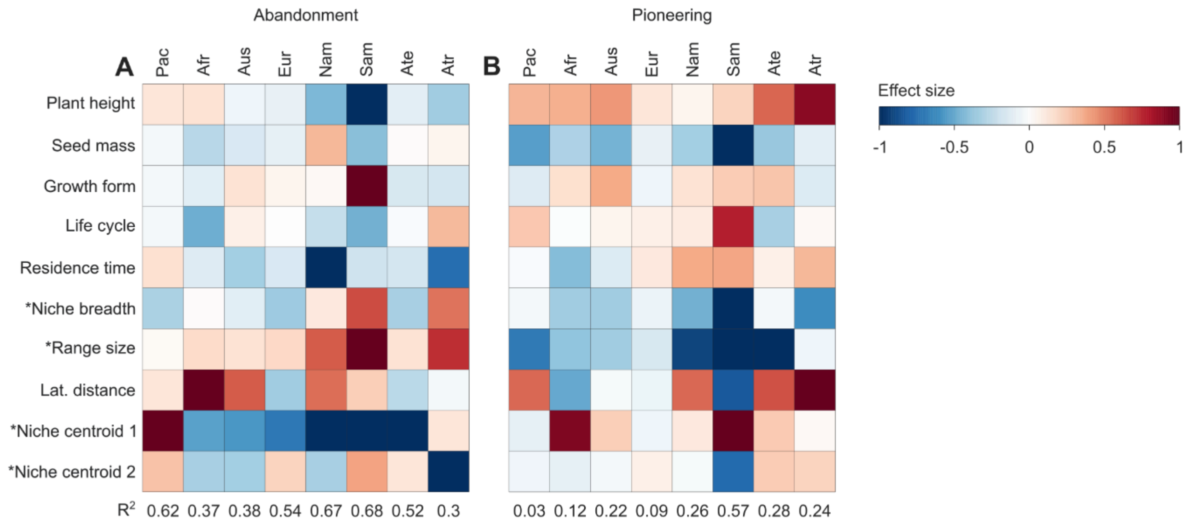

Figure S11 – Trait effects on the non-analogue niche change metrics in non-native plants. For each region, we ran phylogenetic regression models with (A) niche abandonment and (B) niche pioneering as responses. Each column shows the full regional model, with the total explained variance (R^2) of the models shown below the columns. The effect size shows whether the respective niche change metrics will increase (red) or decrease (blue) as the trait values increase. Traits labelled with an asterisk refer to biogeographic traits estimated for the native range or native niche of the species. Niche centroids refer to the relative position along climatic gradient 1 (from warm to cold) and 2 (from wet to dry). The species sample size varied between regions: Africa (Afr, n = 124), temperate Asia (Ate, n = 95), tropical Asia (Atr, n = 78), Australasia (Aus, n = 124), Europe (Eur, n = 56), North America (Nam, n = 110), Pacific Islands (Pac, n = 143), South America (Sam, n = 41). Source data are provided as a Source Data file.

Trait analysis: univariate models for all niche metrics (Figure S12)

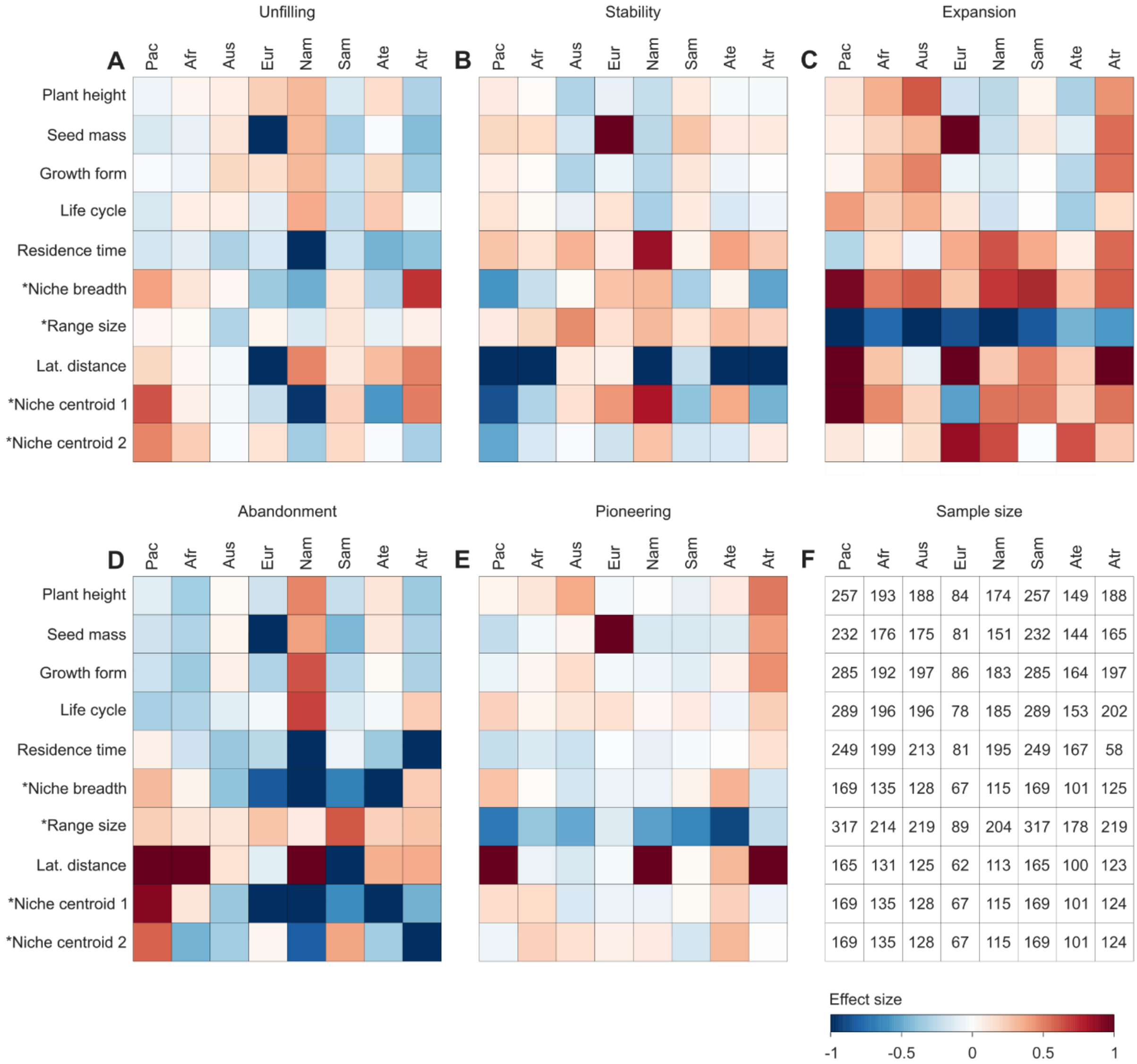

	Pac	Afr	Aus	Eur	Nam	Sam	Ate	Atr
Plant height	257	193	188	84	174	257	149	188
Seed mass	232	176	175	81	151	232	144	165
Growth form	285	192	197	86	183	285	164	197
Life cycle	289	196	196	78	185	289	153	202
Residence time	249	199	213	81	195	249	167	58
*Niche breadth	169	135	128	67	115	169	101	125
*Range size	317	214	219	89	204	317	178	219
Lat. distance	165	131	125	62	113	165	100	123
*Niche centroid 1	169	135	128	67	115	169	101	124
*Niche centroid 2	169	135	128	67	115	169	101	124

Figure S12 – Trait effects on the niche change metrics in non-native plants. For each region, we ran univariate phylogenetic regression models with (A) niche unfilling, (B) niche stability, (C) niche expansion, (D) niche abandonment and (E) niche pioneering as responses. The circle color in each cell indicates the effect size for the predictor corresponding to that row and the region corresponding to that column: The effect size shows whether the respective niche change metrics will increase (red) or decrease (blue) as the trait value increases in that region. Traits labelled with an asterisk refer to biogeographic traits estimated for the native range or native niche of the species. Niche centroids refer to the relative position along climatic gradient 1 (from warm to cold) and 2 (from wet to dry). Note that sample size varies across species and trait combinations (F). Source data are provided as a Source Data file.

Supplementary Tables

ANOVA table for the comparison of the regional niche dynamics (Table S1)

Table S1 – Mean values and ANOVA table for niche unfilling, niche stability, and niche expansion. The intercept always refers to the mean niche metric of the Pacific Islands, and the estimates for the other regions refer to the difference between group-level mean and Pacific Island mean. P-values indicate whether the estimates are significantly different from zero (two-sided t-test). Source data are provided as a Source Data file.

Niche change metric	Region	Mean	ANOVA Estimate	ANOVA Std. Error	ANOVA p-value
Unfilling	Pacific Islands (Intercept)	0.34	-0.65	0.12	5.08e-08 ***
	Africa	0.12	-1.95	0.24	5.20e-08 ***
	temperate Asia	0.25	-1.12	0.21	0.026424 *
	tropical Asia	0.19	-1.44	0.24	0.000973 ***
	Australasia	0.22	-1.26	0.20	0.002216 **
	Europe	0.22	-1.26	0.29	0.035121 *
	North America	0.42	-0.98	0.20	0.088096
	South America	0.19	-1.45	0.21	0.000117 ***
Stability	Pacific Islands (Intercept)	0.61	0.45	0.12	0.000104 ***
	Africa	0.84	1.63	0.22	6.05e-08 ***
	temperate Asia	0.71	0.88	0.20	0.034525 *
	tropical Asia	0.75	1.08	0.22	0.004102 **
	Australasia	0.76	1.14	0.20	0.000430 ***
	Europe	0.74	1.02	0.28	0.037462 *
	North America	0.88	0.86	0.19	0.032844 *
	South America	0.77	1.20	0.20	0.000135 ***
Expansion	Pacific Islands (Intercept)	0.05	-3.04	0.27	<2e-16 ***
	Africa	0.04	-3.21	0.45	0.692
	temperate Asia	0.05	-3.00	0.45	0.942
	tropical Asia	0.06	-2.74	0.44	0.496
	Australasia	0.02	-3.77	0.53	0.165
	Europe	0.04	-3.09	0.60	0.932
	North America	0.03	-3.66	0.53	0.238
	South America	0.04	-3.15	0.43	0.800

Phylogenetic signals in the trait analyses (Table S2)

Table S2 – Phylogenetic signal in the regional trait analyses for all niche metrics, both for the step-selected models and the full models. Source data are provided as a Source Data file.

Model types and niche metrics	Pac	Afr	Aus	Eur	Nam	Sam	Ate	Atr
step-selected models								
unfilling	0	NA	0.062	0	0	0	0	0
stability	0	0.167	0	0	0	0	0.21	0
expansion	0	0.096	0	0.432	0	0	0	0.103
abandonment	0	0.263	0	0	0	0	0.074	0
pioneering	0.576	0.13	0	0.951	0	0	0.094	0
full models								
unfilling	0	0.238	0.11	0	0	0	0	0
stability	0	0.159	0.003	0	0	0	0.217	0
expansion	0	0.109	0	0.459	0	0	0	0.226
abandonment	0	0.243	0	0	0	0	0.138	0
pioneering	0.551	0.058	0	0.962	0	0	0.111	0

R packages used for the analyses

We used R version 4.2.2 for all analyses and visualization, utilizing the following packages:

ade4 (version 1.7-22) [4–8]

BIEN (version 1.26)[9]

conflicted (version 1.2.0.9000)[10]

CoordinateCleaner (version 3.0.1)[11]

corrplot (version 0.92)[12]

devtools (version 2.4.5)[13]

doParallel (version 1.0.17)[14]

dotwhisker (version 0.7.4)[15]

dplyr (version 1.1.2)[16]

ecospat (version 4.0.0)[17]

fasterize (version 1.0.4)[18]

fmsb (version 0.7.6)[19]

foreach (version 1.5.2)[20]

furrr (version 0.3.1)[21]

ggplot2 (version 3.5.1)[22]

GIFT (version 1.0.0)[23]

hrbrthemes (version 0.8.7)[24]

lcvplants (version 2.1.0)[25]

maps (version 3.4.1)[26]

networkD3 (version 0.4)[27]

phylolm (version 2.6.2)[28]

purrr (version 1.0.1)[29]

RColorBrewer (version 1.1-3)[30]

rgbif (version 3.7.5)[31,32]

rWCVP (version 1.2.4)[33]

sf (version 1.0-16)[34,35]

sfheaders (version 0.4.2)[36]

stringr (version 1.5.0)[37]

taxize (version 0.9.100)[38,39]

terra (version 1.7-78)[40]

tibble (version 3.2.1)[41]

tidyr (version 1.3.0)[42]

tidyverse (version 2.0.0)[43]

units (version 0.8-5)[44]

viridis (version 0.6.3)[45]

References

1. Brummitt, R. K., Pando, F. & Hollis, S. *World Geographical Scheme for Recording Plant Distributions*. vol. 951 (International working group on taxonomic databases for plant sciences (TDWG), 2001).
2. Wohlwend, M. *et al.* Data Descriptor: Pacific Introduced Flora (PaciFLora). *BDJ* **9**, e67318 (2021).
3. Köppen, W. The thermal zones of the Earth according to the duration of hot, moderate and cold periods and to the impact of heat on the organic world. *metz* **20**, 351–360 (2011).
4. Dray, S. & Dufour, A.-B. The ade4 Package: Implementing the Duality Diagram for Ecologists. *Journal of Statistical Software* **22**, 1–20 (2007).
5. Bougeard, S. & Dray, S. Supervised Multiblock Analysis in R with the ade4 Package. *Journal of Statistical Software* **86**, 1–17 (2018).
6. Chessel, D., Dufour, A.-B. & Thioulouse, J. The ade4 Package – I: One-Table Methods. *R News* **4**, 5–10 (2004).
7. Thioulouse, J. *et al. Multivariate Analysis of Ecological Data with Ade4*. (Springer, 2018). doi:10.1007/978-1-4939-8850-1.
8. Dray, S., Dufour, A.-B. & Chessel, D. The ade4 Package – II: Two-Table and K-Table Methods. *R News* **7**, 47–52 (2007).
9. Maitner, B. BIEN: Tools for Accessing the Botanical Information and Ecology Network Database. R package version 1.2.6. https://CRAN.R-project.org/package=BIEN (2023).
10. Wickham, H. conflicted: An Alternative Conflict Resolution Strategy. 1.2.0 https://doi.org/10.32614/CRAN.package.conflicted (2018).

11. Zizka, A. *et al.* CoordinateCleaner: Standardized cleaning of occurrence records from biological collection databases. *Methods Ecol Evol* **10**, 744–751 (2019).

12. Wei, T. & Simko, V. *R Package ‘Corrplot’: Visualization of a Correlation Matrix*. (2021).

13. Wickham, H., Hester, J., Chang, W. & Bryan, J. *Devtools: Tools to Make Developing R Packages Easier*. (2022). doi:10.32614/CRAN.package.devtools.

14. Corporation, M. & Weston, S. *doParallel: Foreach Parallel Adaptor for the ‘parallel’ Package*. (2022). doi:10.32614/CRAN.package.doParallel.

15. Solt, F. & Hu, Y. *Dotwhisker: Dot-and-Whisker Plots of Regression Results*. (2021). doi:10.32614/CRAN.package.dotwhisker.

16. Wickham, H., François, R., Henry, L., Müller, K. & Vaughan, D. *Dplyr: A Grammar of Data Manipulation*. (2023).

17. Broennimann, O., Cola, V. D. & Guisan, A. *Ecospat: Spatial Ecology Miscellaneous Methods*. (2023). doi:10.32614/CRAN.package.ecospat.

18. Ross, N. *Fasterize: Fast Polygon to Raster Conversion*. (2022).

19. Nakazawa, M. *Fmsb: Functions for Medical Statistics Book with Some Demographic Data*. (2024). doi:10.32614/CRAN.package.fmsb.

20. Microsoft & Weston, S. *Foreach: Provides Foreach Looping Construct*. (2022).

21. Vaughan, D. & Dancho, M. *Furrr: Apply Mapping Functions in Parallel Using Futures*. (2022).

22. Wickham, H. *Ggplot2: Elegant Graphics for Data Analysis*. (Springer-Verlag New York, 2016).

23. Weigelt, P. & Denelle, P. *GIFT: Access to the Global Inventory of Floras and Traits (GIFT)*. (2023).

24. Rudis, B. *Hrbrthemes: Additional Themes, Theme Components and Utilities for ‘Ggplot2’*. (2024).

25. Freiberg, M. *et al.* LCVP, The Leipzig catalogue of vascular plants, a new taxonomic reference list for all known vascular plants. *Sci Data* **7**, 416 (2020).

26. Becker, O. S. code by R. A., Minka, A. R. W. R. version by R. B. E. by T. P. & team, A. D. F. by the C. *Maps: Draw Geographical Maps*. (2023).

27. Allaire, J. J., Gandrud, C., Russell, K. & Yetman, C. J. *networkD3: D3 JavaScript Network Graphs from R*. (2017).

28. Ho, L. S. T. & Ane, C. A linear-time algorithm for Gaussian and non-Gaussian trait evolution models. *Systematic Biology* **63**, 397–408 (2014).

29. Wickham, H. & Henry, L. *Purrr: Functional Programming Tools*. (2023).

30. Neuwirth, E. *RColorBrewer: ColorBrewer Palettes*. (2022).

31. Chamberlain, S. *et al. Rgbif: Interface to the Global Biodiversity Information Facility API*. (2025).

32. Chamberlain, S. & Boettiger, C. R Python, and Ruby clients for GBIF species occurrence data. *PeerJ PrePrints* https://doi.org/10.7287/peerj.preprints.3304v1 (2017).

33. Brown, M. J. M. *et al.* rWCVP: A companion R package to the World Checklist of Vascular Plants. *New Phytologist* (2023).

34. Pebesma, E. & Bivand, R. *Spatial Data Science: With Applications in R*. (Chapman and Hall/CRC, 2023). doi:10.1201/9780429459016.

35. Pebesma, E. Simple Features for R: Standardized Support for Spatial Vector Data. *The R Journal* **10**, 439–446 (2018).

36. Cooley, D. *Sfheaders: Converts Between R Objects and Simple Feature Objects*. (2023).

37. Wickham, H. *Stringr: Simple, Consistent Wrappers for Common String Operations*. (2022).

38. Chamberlain, S. *et al. Taxize: Taxonomic Information from around the Web*. (2020).

39. Scott Chamberlain & Eduard Szocs. taxize - taxonomic search and retrieval in R. *F1000Research* https://f1000research.com/articles/2-191/v2 (2013).

40. Hijmans, R. J. *Terra: Spatial Data Analysis*. (2024).

41. Müller, K. & Wickham, H. *Tibble: Simple Data Frames*. (2023).

42. Wickham, H., Vaughan, D. & Girlich, M. *Tidyr: Tidy Messy Data*. (2023).

43. Wickham, H. *et al.* Welcome to the tidyverse. *Journal of Open Source Software* **4**, 1686 (2019).

44. Pebesma, E., Mailund, T. & Hiebert, J. Measurement Units in R. *R Journal* **8**, 486–494 (2016).

45. Garnier *et al. Viridis(Lite) - Colorblind-Friendly Color Maps for R*. (2023). doi:10.5281/zenodo.4679424.
